# Descriptive characterization of the 2010 cholera outbreak in Nigeria

**DOI:** 10.1186/1471-2458-14-1167

**Published:** 2014-11-16

**Authors:** Mahmood Muazu Dalhat, Aisha Nasiru Isa, Patrick Nguku, Sani-Gwarzo Nasir, Katharina Urban, Mohammed Abdulaziz, Raymond Salanga Dankoli, Peter Nsubuga, Gabriele Poggensee

**Affiliations:** Nigeria Field Epidemiology and Laboratory Training Programme, Abuja, Nigeria; Department of Public Health, Federal Ministry of Health, Abuja, Nigeria; Global Public Health Solutions, Atlanta, GA USA

**Keywords:** Cholera, Outbreak, Nigeria, Case fatality rate, Attack rate

## Abstract

**Background:**

In 2010, 18 States of Nigeria reported cholera outbreaks with a total of 41,787 cases including 1,716 deaths (case-fatality rate [CFR]: 4.1%). This exceeded the mean overall CFR of 2.4% reported in Africa from 2000–2005 and the WHO acceptable rate of 1%. We conducted a descriptive analysis of the 2010 cholera outbreak to determine its epidemiological and spatio-temporal characteristics.

**Methods:**

We conducted retrospective analysis of line lists obtained from 10 of the 18 states that submitted line lists to the Federal Ministry of Health (FMOH). We described the outbreak by time, place and person and calculated the attack rates by state as well as the age- and sex-specific CFR from cholera cases for whom information on age, sex, place of residence, onset of symptoms and outcome were available.

**Results:**

A total of 21,111 cases were reported with an overall attack rate and CFR of 47.8 cases /100,000 population and 5.1%, respectively. The CFR ranged in the states between 3.8% and 8.9%. The age-specific CFR was highest among individuals 65 years and above (14.6%). The epidemiological curve showed three peaks with increasing number of weekly reported cases. A geographical clustering of LGAs reporting cholera cases could be seen in all ten states. During the third peak which coincided with flooding in five states the majority of newly affected LGAs were situated next to LGAs with previously reported cholera cases, only few isolated outbreaks were seen.

**Conclusion:**

Our study showed a cholera outbreak that grew in magnitude and spread to involve the whole northern part of the country. It also highlights challenges of suboptimal surveillance and response in developing countries as well as potential endemicity of cholera in the northern part of Nigeria. There is the need for a harmonized, coordinated approach to cholera outbreaks through effective surveillance and response with emphasis on training and motivating front line health workers towards timely detection, reporting and response. Findings from the report should be interpreted with caution due to the high number of cases with incomplete information, and lack of data from eight states.

## Background

Cholera is an acute enteric infection caused by the bacterium *Vibrio (V.) cholerae* of serogroup O1 or O139*.* It is a waterborne disease of important public health importance with an estimated number of 3 to 5 million cases annually and 100,000 to 150, 000 deaths yearly [1]. Outbreaks are linked to the consumption of unsafe water and food, poor hygiene and sanitation. Cholera often follows natural or man-made disasters which can lead to internal displacement of persons and subsequent unstable living conditions associated with contamination of food and water sources [2, 3]. Overflowing of latrines and contamination of wells and surface water, seasonal modification of water sources for consumption and human behavior may play a role in the occurrence of cholera outbreaks [4]. Control of cholera outbreaks requires effective surveillance and response systems which are frequently sub-optimal in developing countries often lacking robust data collection, collation, analysis, interpretation and response [5]. Poor detection and delayed response to cholera outbreaks can result in geographical spread of the disease and consequently high attack rates and case fatality rates [6–8]. Failure to control local outbreaks and prevention of between-region transmission could result in spread of cholera outbreaks to neighboring regions [9].

In 2010, a total of 110,115 cases were reported from sub-Saharan African countries, a 46% decline compared to 2009. However, four countries in Central Africa around the Lake Chad Basin (Cameroon, Chad, Niger and Nigeria) accounted for 62,762 cases, including 2,610 deaths, i.e. 54% of cases and 77% of deaths reported from the continent. Nigeria reported 38% of cases from Africa with 44,456 cases and a case fatality rate (CFR) of 3.9% [10]. This clearly exceeds the mean overall CFR of 2.4% reported in Africa from 2000–2005 and by far, the WHO acceptable rate of 1%. The 2010 cholera outbreak was the largest epidemic in Nigeria since 1991 when 59,478 cases and 7,654 deaths were reported [11]. The outbreak started from north-eastern border state of Borno and spread to involve 18 of the 36 states of the country. We conducted a descriptive analysis of surveillance data of the 2010 cholera outbreak with the aim of determining the epidemiological and spatio-temporal characteristics to explore possible reasons for the nationwide spread so as to provide information for future response to cholera outbreaks.

## Methods

**Figure 1:**
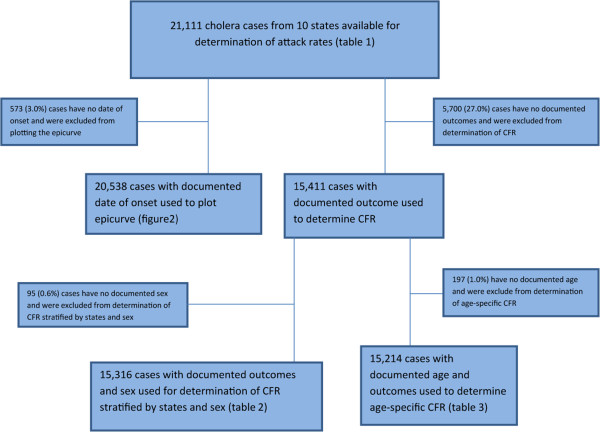
**Flow chart of cholera cases reported in 2010 included in the analyses.**

We conducted a retrospective analysis of cholera cases with information on age, sex, place of residence (Local Government Area (LGA) and onset of symptoms. All state epidemiologists who reported outbreaks to the Federal Ministry of Health (FMOH) were requested to send copies of their line lists. In cases where NFELTP residents were sent for outbreak investigations, they retrieved and submitted the line lists for analysis. Data were extracted from the line lists obtained from 10 of the 18 states that submitted line lists to the FMOH: Adamawa, Bauchi, Borno, Gombe, Jigawa, Kaduna, Katsina, Sokoto, Taraba, and Yobe. A cholera case was defined as any patient with acute watery diarrhea, with or without vomiting, during the period of the outbreak, based on the case definition provided in the National Technical Guidelines of the Integrated Diseases Surveillance and Response (IDSR) [12]. We included cases under the age of 5 years in our analysis as is been done in outbreak settings [7, 13, 14]. We analyzed and characterized the data in time, place, and person using Epi Info version 3.5.3. We computed the attack rate (AR: cases/100,000 population) using reported cases and the projected population data based on the 2006 census (Table 1) [15]. Case fatality rate (CFR) and age-specific CFR were calculated using cases with complete clinical information, which we defined as any patient with data on age, sex, state, and outcome (Figure 1). The numerator for the age-specific CFR is the number of deaths in the specific age group, while the denominator was the number of cases in the specific age groups with complete clinical information. All cases with incomplete information were excluded from the determination of CFR. We defined the first epidemiological week of the year as the week that ends on the first Saturday of January, as long as it falls at least 4 days into the month. Subsequently each epidemiological week begins on a Sunday and ends on a Saturday. For analysis, the year 2010 was divided into time periods (“waves”). A wave was defined as the time period from the beginning of a peak (first epidemiologic week with rising numbers of reported cases) to the end of a peak (epidemiologic week with nadir of reported cases/week before the next rise). The time periods were defined as follows: wave 1: epidemiological week 1 – 9; wave 2: epidemiologic week 10 – 24; wave 3: epidemiologic week 25 – 42.

**Table 1:** **Cholera attack rates stratified by states, Nigeria 2010**

State	Cases	Projected 2010 population	Attack rate/100,000 population
Adamawa	2,004	3,569,948	56,1
Bauchi	4,660	5,330,933	87,4*
Borno	2,616	4,778,758	54,7
Gombe	1,089	2,687,993	40,5
Jigawa	741	4,897,387	15,1*
Kaduna	778	6,892,955	11,3
Katsina	5,704	6,541,267	87,2*
Sokoto	248	4,174,756	5,9*
Taraba	536	2,577,051	20,8*
Yobe	2,735	2,670,175	102,4
Total	21,111	44,121,223	47,8

*States where flooding occurred between August and October 2010.

For the statistical analysis, we used the chi square test and compared medians to determine the difference in age groups affected during the different waves.

### Ethical considerations

The surveillance data used for this analysis were generated within the frame of the IDSR in Nigeria [12] and analysed as part of an outbreak response of state and federal public health officials and residents of the NFELTP. Ethical waiver for the conduct of the study was obtained from the ethical committee of Aminu Kano Teaching Hospital, Kano, Nigeria. The information collected in the data set (age, sex, date of onset of illness, Local government area, and outcome) was safeguarded within the database of the programme.

## Results

**Figure 2:**
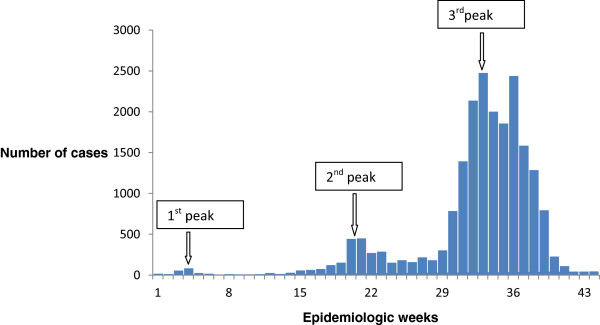
**Epicurve of the cholera outbreak 2010, Nigeria.**

We identified 21,111 cases (50.5% of all reported cases) that met the case definition among a total population of 44,121,223 in the 10 states. The first cases were documented in Borno State on 2nd January, 2010. The epidemic curve showed three peaks (Figure 2). The first peak was seen in week 4 (85 cases/week; cases reported from Borno and Yobe State) and the second peak was observed in week 21 (444 cases/week) with cases reported from five states. The third, most pronounced peak was observed in week 33 with all the 10 states affected (2,476 cases/week). Thereafter, with exception of week 36, the outbreak declined. By the epidemiological week 43 only 3 cases were reported.

**Figure 3:**
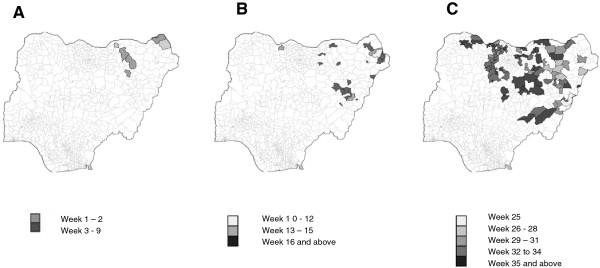
**Epidemiologic week of first reported cases in the Local Government areas during the three waves (Wave 1 (A): week 1 – week 9; wave 2 (B): week 10 – week 24; wave 3 (C): week 25 – week 42).**

Overall, a geographical clustering of LGAs reporting cholera cases could be seen in all 10 states. The temporal spread of cholera as shown in Figure 3 revealed that only three LGAs were geographically isolated; the majority of LGAs were contiguous with more than one LGA reporting cholera cases (Figure 3). In the first wave with a maximum of less than 100 new cases per week, two LGAs in the east of Borno State situated at the border to Chad and Niger and five LGAs in Yobe State were affected. During the second wave with a peak of more than 400 new cases per week, 5 LGAs neighboring the first wave LGAs having reported cholera cases. Furthermore a new cluster was seen in Gombe State and several isolated outbreaks occurred in Jigawa and Katsina State. During the third wave, which coincided with flooding in five states and had the highest number of reported new cases per week, the majority of newly affected LGAs were situated next to LGAs with previously reported cholera cases, only few isolated outbreaks were seen.

**Figure 4:**
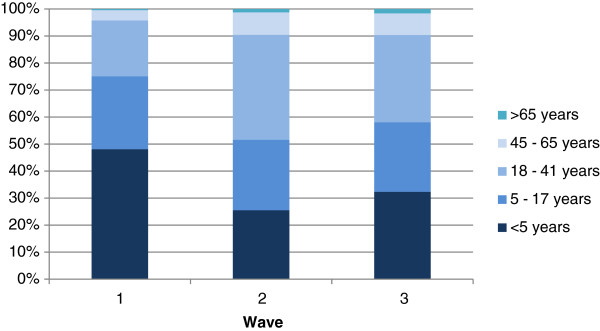
**Age distribution of cholera cases during the three waves (wave 1: week 1 – 9; wave 2: week 10 – 24; wave 3: week 25 – 42), Nigeria, 2010.**

The median age of cases was 12 years (Interquartile range (IQR): 3–17), 51.5% were female (10,651 out of 20,688). The median age during the three waves differed significantly (p <0.001): wave 1: 6 years (IQR: 5–29 years), wave 2: 17 years (IQR: 5–29 years), wave 3: 12 years (IQR: 4–27 years). Children younger than 5 years were the most affected group in the first wave (40.9%), whereas in the second and third wave cholera cases were more frequently reported in adults aged 17 – 55 years (46.1% and 39.1%, respectively; Figure 4).

The highest absolute numbers of cholera cases were seen in Bauchi and Katsina States (Table 1). The overall cholera attack rate (AR) in the 10 states was 47.8/100,000 population.

For 15,316 cases (72.5%), adequate clinical information was available for determination of CFR (Figure 1). A total of 784 deaths were documented given an overall CFR of 5.1% (Table 2). The CFR ranged in the states between 3.8% and 8.9%. The age-specific case fatality rate was highest among the elderly (65 years and above) with 14.5% (Table 3).

**Table 2:** **Case fatality rates of cholera outbreak 2010 stratified by states and sex, Nigeria 2010 (n =15,316)**

State	Female				Male				Overall CFR (%)
	Total number of cases	Alive	Dead	CFR (%)	Total number of cases	Alive	Dead	CFR (%)	
Adamawa	1,054	994	60	5.7	948	885	63	6.6	6.1
Borno	1,188	1,105	83	7.0	1,314	1,246	68	5.2	6.0
Gombe	544	527	17	3.1	516	493	23	4.5	3.8
Jigawa	369	349	20	5.4	358	343	15	4.2	4.8*
Kaduna	385	365	20	5.2	393	373	20	5.1	5.1
Katsina	2,827	2,720	107	3.8	1,903	1,810	93	4.9	4.2
Sokoto	131	118	13	9.9	117	108	9	7.7	8.9*
Taraba	240	222	18	7.5	296	277	19	6.4	6.9*
Yobe	1,259	1,208	51	4.1	1,474	1,389	85	5.8	5.0
Total	7,997	7,608	389	4.9	7,319	6,924	395	5.4	5.1

*States with cholera camps during the outbreak.

**Table 3:** **Age-specific case fatality rates of cholera cases, Nigeria 2010**

Age group (yrs)	Cases with documented outcome	Outcomes		Age-specific case fatality rates (%)
		Alive	Dead	
<5	3,940	3,788	152	3.9
5 – 17	4,680	4,443	237	5.1
18 – 44	5,015	4,792	223	4.4
45 – 64	1,282	1,160	122	9.5
65 and above	297	254	43	14.5
Total	15,214	14,437	777	5.1

## Discussion

Our descriptive study analyzed a large cholera epidemic that spanned most of the year 2010 based on data from ten out of 18 affected states of Nigeria. The epidemic started in local government areas in the eastern part of the country with two local government areas neighboring an international border (Chad and Niger). Eventually the outbreak spread throughout the northern part of Nigeria in two more waves, the last wave with nearly 2,500 new cases per week with case fatality rates up to 8%.

High case fatality rates seen in outbreaks are associated with limited access to health care, insufficiencies of the health care system and limitations in the surveillance system capacities to trigger timely response [16]. With proper and timely case management a CFR of less than 1% can be achieved [17]. In 2010, Nigeria had a CFR of 5.1%, the highest in Africa. The CFR in the elderly (65 years and above) was significantly higher compared to the other age groups. Limited access to health services due to flooding and subsequent displacement during the third wave might have been a contributing factor; furthermore the high CFR might have been associated with co-morbidities in this age group. Marin et al. have shown that the cholera outbreaks in 2009 and 2010 in Nigeria were caused by multidrug resistant atypical El Tor O1 strains, which are reportedly highly virulent [18]. They concluded that guidelines for managing and containing cholera outbreaks in Nigeria (which include, in addition to rehydration, using the antimicrobials trimethoprim, and more recently ciprofloxacin) need to be revised to reflect local antimicrobial susceptibility testing in line with recent findings and universal guidelines [19–22].

Generally, children younger than 5 years have the highest incidence of cholera and the age-specific mortality is highest in this age group [13, 14]. However, during the 2010 outbreak in Nigeria a different pattern was seen; only during the first wave children below the age of 5 years were the most affected group, during the second and third wave the majority of cases were adults. A possible explanation might have been increased exposure to *V. cholera*e due to the displacement of inhabitants associated with limited access to safe water during the floodings.

The outbreak appeared to have spread from Borno State to other states of the north-eastern region. These are states where people travel to and fro within a day. It is noteworthy that the outbreak had three waves with progressively increasing numbers with at least 12 weeks in between the waves. Our findings indicated that cases reported during the second and third waves of the cholera outbreak were mainly living in LGAs contiguous with areas that had previously reported cholera outbreaks during the first wave. An effective response to the outbreak in Borno therefore might have prevented the spread of cholera to other parts of the country. End of August 2010, during the rainy season (March to September) flooding occurred in some northern parts of Nigeria. As of October 2010 Jigawa, Katsina and Sokoto States were affected, and it was estimated that about 258,000 inhabitants were displaced due to the floods [23, 24]. Limited access to safe water resulting in contaminated food, insufficient sanitation and limited access to health services due to displacement might have contributed to the spread and impeded the containment of the outbreaks in these states during the third wave as established in other settings [6–8, 25].

Cholera cases were recorded from the beginning to the end of the outbreak in Borno State, suggesting possible endemicity of cholera in the country. This is in keeping with previous findings by Gaffga et al. and Maramovich and Deen in their separate reviews of cholera in Africa [26–28].

The study highlights the challenges faced by developing countries in creating effective surveillance as well as preparedness and response to cholera outbreaks. There is the need for an effective surveillance system with the capacity to timely and appropriately respond to and contain cholera outbreaks locally before they spread to neighboring areas. The amount of data missing in the evaluation and the fact that cases reported from a whole state had no documented outcome implies that important epidemiological variables like the AR and CFR could not be accurately determined. The high number of children under 5 years documented during the outbreak indicates that the current IDSR definition that excludes children under 5 years should be reviewed for countries endemic for cholera or during outbreaks [27]. This fact has been considered by Heymann in his case definition of cholera [17].

Our findings should be interpreted with caution due to the high number of cases with incomplete information, and lack of data from eight states. This could affect the accuracy of the estimated AR and CFR as well as limit the ability to generalize our findings. The absence of data on clinical management also limits our ability to identify poor clinical case management as a cause of high CFR.

## Conclusions

Our study showed a cholera outbreak that grew in magnitude and spread to involve the whole northern part of the country. It also showed potential endemicity of cholera in the northern part of Nigeria. There is the need for a harmonized, coordinated approach to cholera outbreaks through effective surveillance and response with emphasis on training and motivating front line health workers towards timely detection and response as well as proper documentation.
